# Alpha-fetoprotein combined with initial tumor shape irregularity in predicting the survival of patients with advanced hepatocellular carcinoma treated with immune-checkpoint inhibitors: a retrospective multi-center cohort study

**DOI:** 10.1007/s00535-024-02202-y

**Published:** 2024-12-23

**Authors:** Feng Zhang, Yong-Shuai Wang, Shao-Peng Li, Bin Zhao, Nan Huang, Rui-Peng Song, Fan-Zheng Meng, Zhi-Wen Feng, Shen-Yu Zhang, Hua-Chuan Song, Xiao-Peng Chen, Lian-Xin Liu, Ji-Zhou Wang

**Affiliations:** 1 Department of Hepatobiliary Surgery, Centre for Leading Medicine and Advanced Technologies of IHM, The First Affiliated Hospital of USTC, Division of Life Sciences and Medicine, University of Science and Technology of China, Hefei, Anhui 230001 China; 2 Anhui Provincial Key Laboratory of Hepatopancreatobiliary Surgery, Hefei, Anhui 230001 China; 3 Anhui Provincial Clinical Research Center for Hepatobiliary Diseases, Hefei, Anhui 230001 China; 4 Department of Radiology, The First Affiliated Hospital of USTC, Division of Life Sciences and Medicine, University of Science and Technology of China, Hefei, Anhui 230001 China; 5 Department of Hepatobiliary Surgery, The First Affiliated Hospital of Wannan Medical College (Yijishan Hospital of Wannan Medical College), Wuhu, Anhui 241000 China

**Keywords:** Immune checkpoint inhibitor, Prognosis, Hepatocellular carcinoma, Alpha-fetoprotein, Tumor shape irregularity

## Abstract

**Background:**

Immune checkpoint inhibitors (ICIs) are playing a significant role in the treatment of hepatocellular carcinoma (HCC). This study aims to explore the prognostic value of alpha-fetoprotein (AFP) and initial tumor shape irregularity in patients treated with ICIs.

**Methods:**

In this retrospective, multi-center study, 296 HCC patients were randomly divided into the training set and the validation set in a 3:2 ratio. The training set was used to evaluate prognostic factors and to develop an easily applicable ATSI (AFP and Tumor Shape Irregularity) score, which was verified in the validation set.

**Results:**

The ATSI score was developed from two independent prognostic risk factors: baseline AFP ≥ 400 ng/ml (HR 1.73, 95% CI 1.01–2.96, *P* = 0.046) and initial tumor shape irregularity (HR 1.94, 95% CI 1.03–3.65, *P* = 0.041). The median overall survival (OS) was not reached (95% CI 28.20–NA) in patients who met no criteria (0 points), 25.8 months (95% CI 14.17–NA) in patients who met one criterion (1 point), and 17.03 months (95% CI 11.73–23.83) in patients who met two criteria (2 points) (*P* = 0.001). The median progression-free survival (PFS) was 10.83 months (95% CI 9.27–14.33) for 0 points, 8.03 months (95% CI 6.77–10.57) for 1 point, and 5.03 months (95% CI 3.83–9.67) for 2 points (*P* < 0.001). The validation set effectively verified these results (median OS, 37.43/24.27/14.03 months for 0/1/2 points, *P* = 0.028; median PFS, 13.93/8.30/4.90 months for 0/1/2 points, *P* < 0.001).

**Conclusions:**

The ATSI score can effectively predict prognosis in HCC patients receiving ICIs.

**Supplementary Information:**

The online version contains supplementary material available at 10.1007/s00535-024-02202-y.

## Introduction

Hepatocellular carcinoma (HCC) is the most common primary liver cancer with high malignancy, frequently relapse, and unfavorable prognosis [1]. Over the past decade, sorafenib has been the first-line treatment for advanced HCC patients in the Asia–Pacific region and western countries [2, 3]. IMBrave-150 demonstrated that the atezolizumab plus bevacizumab outperformed sorafenib, setting a new first-line standard of systemic treatment [4]. Subsequently, an increasing number of immunological drugs have been developed and manufactured. The immune checkpoint inhibitors (ICIs) show remarkable efficacy and great promise [5, 6]. The treatment of liver cancer has now entered a new era of immunotherapy.

However, immunotherapy can only benefit some patients and the overall response rate remains low, with considerable variation between individuals [7]. It is very important to screen out suitable patients for immunotherapy before treatment. Several predictors of the prognosis of ICIs have been reported, including programmed death ligand 1 (PD-L1) expression levels, high-frequency microsatellite instability/deficient mismatch repair (MSI-H/dMMR), and tumor mutational burden (TMB) [8–12]. But current immunotherapy predictors still have many problems that limit their clinical application, such as: low accuracy, expensive, invasive, complex, and poor clinical utility [13–15]. Simple, reliable, and clinically useful prognostic predictors for immunotherapy are still lacking. The predictive score for screening patients for potential immunotherapy benefits are urgently needed.

The pre-treatment clinical parameters (tumor characteristics, peripheral blood routine indexes, biochemical indexes, etc.) are routine indicators that can be easily accessed in the clinic, providing reliable data on the tumor tissue and the host immune status. Therefore, it is necessary to draw on potential tumor characteristics and indicators that can predict a patient’s reaction to immunotherapy before it is administered.

Alpha-fetoprotein (AFP) is a well-known serum marker commonly used for the diagnosis, monitoring, and prognosis of HCC. Some studies suggest that baseline AFP levels before immunotherapy are related to tumor response and treatment prognosis in HCC [16, 17]. In addition, the imaging features of tumors are utilized to forecast the effectiveness and outcome of treatments. The relevant studies have shown that the irregular shape of tumors is related to poorer survival outcomes in patients with surgically removed HCC [18]. However, the association of tumor shape with immune response and outcomes in HCC patients has not been explored. Based on this, we will investigate the predictive utility of baseline AFP and tumor shape for efficacy and prognosis in HCC patients receiving immunotherapy in this study.

In conclusion, we developed and validated an easily applicable score based on the baseline AFP level and initial tumor shape to predict the prognosis of HCC patients treated with ICIs.

## Methods

### Study design and patients

This study included 395 HCC patients who received ICIs treatment from January 2019 to December 2023 at two centers of the Liver Cancer Clin-Bio Databank of Anhui Hepatobiliary Surgery Union (LCCBD_AHSU) in China. LCCBD_AHSU was a database that was maintained follow-up prospectively and collected data retrospectively. All study procedures were approved by the Institutional Ethics Committee of The First Affiliated Hospital of the University of Science and Technology of China (approval number: 2024KY304). All patients provided either a signed or online consent form.

The diagnostic criteria for HCC are in accordance with the Guidelines for the Diagnosis and Treatment of Hepatocellular Carcinoma (2019 edition) [19]. The inclusion criteria included: (1) confirmed diagnosis of hepatocellular carcinoma; (2) obtainable test and imaging results; (3) assessable tumor lesions; (4) complete follow-up results. The exclusion criteria included: (1) insufficient clinical and follow-up data; (2) history of other cancers; (3) other serious systemic diseases; (4) concurrent infections that could affect the data. 99 patients were excluded due to meeting the exclusion criteria. Finally, 296 HCC patients with complete data were randomized 3:2 into training and validation sets. The training set was used to identify independent risk factors associated with prognosis and then to develop a new simple score based on these factors. The validation set was used to replicate and test the performance of this score. The research process and randomization flowchart were shown in Fig. S1.

The baseline defined as prior to initiating treatment with ICIs. The clinical, pathological, and demographical data were collected and analyzed through the patient’s medical records, including gender, age, hepatitis B, Barcelona Clinic Liver Cancer (BCLC) stage [20], presence of cirrhosis, Child–Pugh Class, Eastern Cooperative Oncology Group Performance Status (ECOG-PS), laboratory test results, and tumor characteristics.

**Fig. 1:**
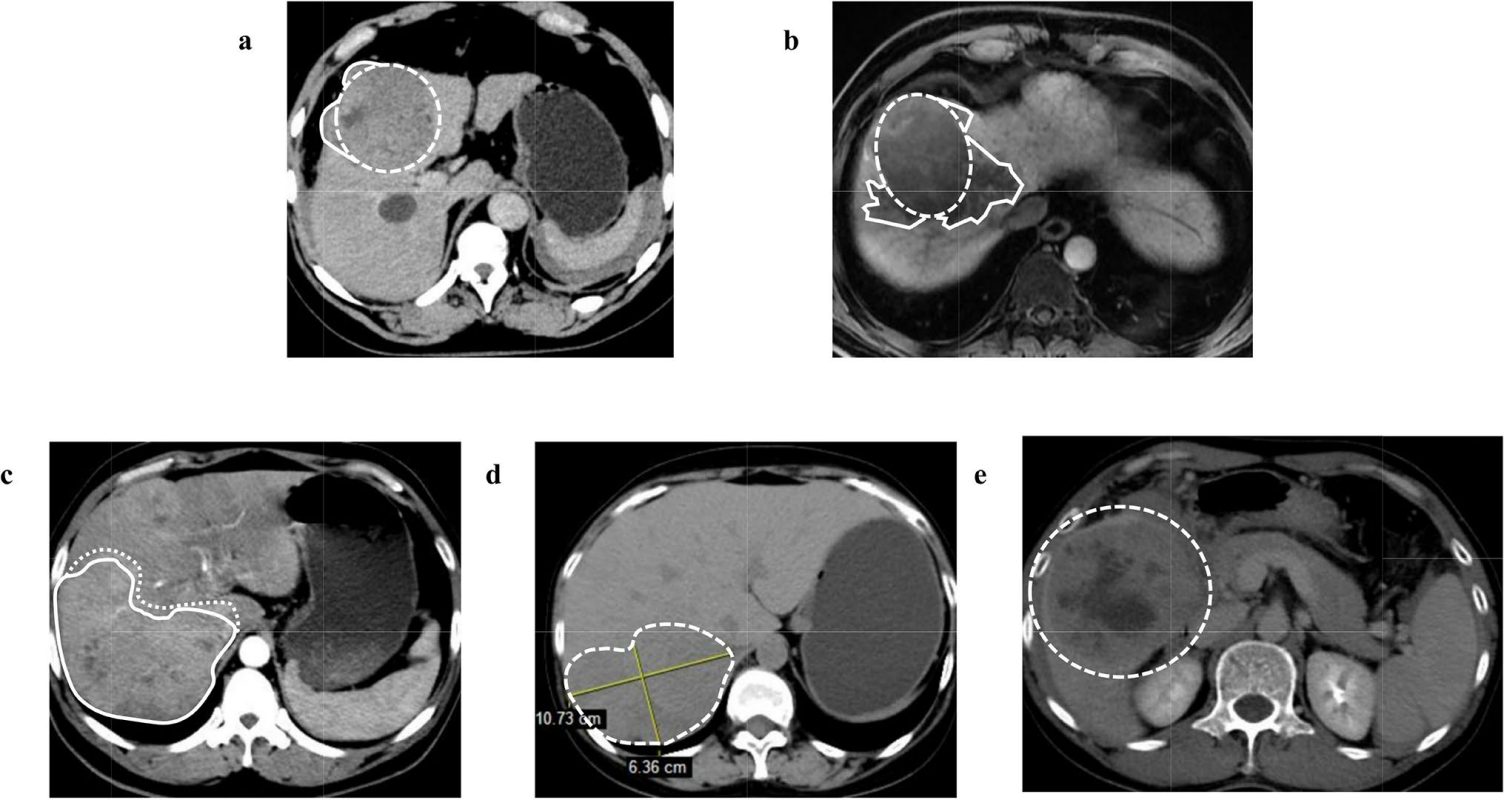
Irregular and regular tumor shape on cross-sectional imaging. **a** The margins of the tumor are smooth but distorted, locally protruding from the tumor, which exhibited a lobulated appearance, and more than two of the tumor margins are at an angle of less than 180°. **b** Non-smooth tumor margins with sharp multilocularity, in which single nodules larger than 0.5 cm or multiple nodules with maximum protrusion larger than 0.3 cm. **c** The margins of the tumor are blurred. The interface between the tumor and liver parenchyma is unclear. **d** The tumor is non-circular or oval, with a long-to-short-axis ratio greater than 1.5, and presents asymmetrically. **e** Tumor with regular shape

The tumor shape was defined as the interface between the tumor and normal hepatic parenchyma, the tumor shape is judged to be irregular if it meets any of the following four criteria on a computed tomography (CT) or magnetic resonance imaging (MRI) scan cross-section (Fig. 1) [21–23]:

1. The margins of the tumor are smooth but distorted, locally protruding from the tumor, which exhibited a lobulated appearance, and more than two of the tumor margins are at an angle of less than 180° (Fig. 1a).
2. Non-smooth tumor margins with sharp nodules, in which single nodules larger than 0.5 cm or multiple nodules with maximum protrusion larger than 0.3 cm (Fig. 1b).
3. The margins of the tumor are blurred. The interface between the tumor and liver parenchyma is unclear (Fig. 1c).
4. The tumor is non-circular or oval, with a long-to-short-axis ratio greater than 1.5, and presents asymmetrically (Fig. 1d).

The regular tumor shape is shown in Fig. 1e. The tumor shape was evaluated separately by three independent radiologists who were blinded to the patients’ conditions. If the patient has multiple tumors of different shapes, it is judged to be irregular as long as one lesion meets any of the above criteria.

### Evaluation on therapeutic outcome and follow-up

The imaging tests such as CT or MRI were used to assess the effectiveness of the treatment, and tumor response was assessed as often as once after receiving four cycles of ICIs treatment. Assess the patient’s response to immunotherapy based on the Response Evaluation Criteria in Solid Tumors version 1.1 (RECIST ver.1.1) [24]. In particular, the results of evaluating effectiveness were classified as complete response (CR), partial response (PR), stable disease (SD), or progressive disease (PD). Survival follow-up was conducted every 3 months after the immunotherapy started.

The progression-free survival (PFS) was defined as the period starting from the patient’s first treatment with immunotherapy until tumor progression, death, or the last follow-up. Overall survival (OS) was defined as the period starting from the patient’s first treatment with immunotherapy until death from any cause or the last follow-up.

### Statistical analyses

The data were analyzed and presented using SPSS 27.0 and GraphPad Prism 8.0 statistical software. Normal distributions were tested for all variables. The variables with normal distribution are presented as mean ± standard deviation (*X* ± *S*), while medians and quartiles (P25, P75) were used for variables that were not normally distributed. The categorical variables are expressed as *n* (%).

The patients were randomly assigned to either the training set or the validation set in a ratio of 3:2. The optimal threshold value of AFP before treatment was calculated using the receiver operating characteristic (ROC) curve. The point with the largest Uden’s index (sensitivity + specificity − 1) was chosen as the best cutoff value point for AFP. The prognostic risk factors explored using Cox proportional hazards regression models. Disparities in survival between patient groups were assessed by univariate analysis of variance using the log-rank test; to exclude the influence of confounding factors, variables with *P* < 0.10 in univariate analysis were incorporated into the multivariate Cox regression analysis to analyze the independent prognostic factors affecting the OS. The Kaplan–Meier method was used to plot survival curves for OS and PFS. Statistical significance was determined at *P* < 0.05 using the bilateral test for all analyses in the study.

## Results

### Patient characteristics

This study included 296 patients with HCC receiving immunotherapy, whose baseline characteristics are detailed in Table 1. In the training set, the median age of patients was 56 years, while in the validation set it was 57 years. Over 88% of individuals in the training group and over 86% of individuals in the validation group were male. The median body mass index (BMI) was 22.49 (20.31, 24.61) kg/m^2^ and 21.47 (20.33, 24.45) kg/m^2^ in the training and validation sets, respectively. The patients with hepatitis B in the training set was 90.40% compared to 88.98% in the validation set. In the training set, there were 133 patients classified as Child–Pugh class A (75.14%) and 44 as class B (24.86%). The validation set included 91 patients classified as A (76.47%), 27 as B (22.69%), and 1 as C (0.84%). In the training and validation groups, 98.87% and 99.16% of patients had an ECOG PS score of 0–1, respectively. 52 (29.38%) of patients had postoperative recurrence and 90 (50.85%) had prior transcatheter arterial chemoembolization (TACE) treatment in the training set, which was not statistically different from the validation set. In the training set, 115 patients (64.97%) received first-line immunotherapy and 62 patients (35.03%) received second or later line immunotherapy; while in the validation set, 71 patients (59.66%) received first-line immunotherapy and 48 patients (40.34%) received second or later line immunotherapy. 41.24% of patients in the training set were treated with a combination of targeted therapy compared to 37.82% in the validation set. In addition, macrovascular invasion was observed in 83 patients (46.89%) in the training set and 55 patients (46.22%) in the validation set. In the training set, extrahepatic spread was present in 81 patients (45.76%), while it was present in 49 patients (41.53%) of the validation set. The BCLC stages for the training and validation sets were as follows: *n* = 9 (5.08%) vs. *n* = 6 (5.04%) for stage 0, *n* = 10 (5.65%) vs. *n* = 9 (7.56%) for stage A, *n* = 26 (14.69%) vs. *n* = 27 (22.69%) for stage B, and *n* = 132 (74.58%) vs. *n* = 77 (64.71%) for stage C, respectively. The baseline characteristics did not show statistically significant variance between patients in the training and validating sets (*P* > 0.05).

**Table 1:** Patient characteristics of the training and validation cohort

Variables	Training set (*n* = 177)	Validation set (*n* = 119)	*P* value
Age, years	56.00 (49.00, 64.00)	57.00 (50.00, 67.00)	0.193
SEX, *n* (%)			0.687
Male	156 (88.14)	103 (86.55)	
Female	21 (11.86)	16 (13.45)	
BMI, kg/m^2^	22.49 (20.31, 24.61)	21.47 (20.33, 24.45)	0.250
Hepatitis B virus, *n* (%)	160 (90.40)	105 (88.98)	0.694
Child–Pugh stage, *n* (%)			0.440
A	133 (75.14)	91 (76.47)	
B	43 (24.86)	27 (22.69)	
C	0 (0.00)	1 (0.84)	
ECOG PS, *n* (%)			0.917
0	67 (37.85)	47 (39.50)	
1	108 (61.02)	71 (59.66)	
2	2 (1.13)	1 (0.84)	
Postoperative recurrence, *n* (%)	52 (29.38)	25 (21.01)	0.108
Prior TACE, *n* (%)	90 (50.85)	53 (44.54)	0.287
The lines of therapy, *n* (%)			0.354
First-line therapy	115 (64.97)	71 (59.66)	
Second-/later line therapy	62 (35.03)	48 (40.34)	
Combined targeted therapy, *n* (%)	73 (41.24)	45 (37.82)	0.555
Tumor number, *n* (%)			0.727
Single	31 (17.51)	19 (15.97)	
Multiple	146 (82.49)	100 (84.03)	
Tumor size, *n* (%)			0.153
< 5 cm	71 (40.11)	38 (31.93)	
≥ 5 cm	106 (59.89)	81 (68.07)	
Lymph node metastasis, *n* (%)	85 (48.02)	51 (42.86)	0.382
Macrovascular invasion, *n* (%)	83 (46.89)	55 (46.22)	0.909
Extrahepatic spread, *n* (%)	81 (45.76)	49 (41.53)	0.473
BCLC stage, *n* (%)			0.269
0	9 (5.08)	6 (5.04)	
A	10 (5.65)	9 (7.56)	
B	26 (14.69)	27 (22.69)	
C	132 (74.58)	77 (64.71)	
Alpha-fetoprotein, *n* (%)			0.621
< 400 ng/ml	105 (59.32)	74 (62.18)	
≥ 400 ng/ml	72 (40.68)	45 (37.82)	
Initial tumor shape, *n* (%)			0.556
Regularity	79 (44.63)	49 (41.18)	
Irregularity	98 (55.37)	70 (58.82)	
AST, IU/l	47.30 (30.00, 72.00)	44.00 (30.15, 87.50)	0.476
ALT, IU/l	33.00 (20.00, 53.00)	31.00 (21.00, 56.30)	0.852
PLT, 10^9^/l	125.50 (86.25, 184.50)	129.00 (80.00, 196.00)	0.470
WBC, 10^9^/l	4.66 (3.55, 6.23)	4.78 (3.34, 6.53)	0.712

*BMI* body mass index, *BCLC* Barcelona-Clinic Liver Cancer, *ECOG PS* Eastern Cooperative Oncology Group Performance Status, *TACE* transcatheter arterial chemoembolization, *ALT* alanine aminotransferase, *AST* aspartate aminotransferase, *PLT* platelet count, *WBC* white blood cell count, *NLR* neutrophil–lymphocyte ratio

The AFP value before immunotherapy was used to create the ROC curve, which determined the optimal cutoff value of 386.43 ng/ml for AFP (Fig. S2). After rounding, the individuals were divided into categories of high and low AFP levels using a cut-off value of 400 ng/ml. 72 patients (40.68%) exhibited AFP levels ≥ 400 ng/ml, and 105 (59.32%) patients exhibited AFP levels < 400 ng/ml. The initial tumor shape was irregular in 98 patients (55.37%) and regular in 79 patients (44.63%).

### Univariate and multivariate Cox regression analyses of OS and PFS in the training set

In the multivariate Cox regression analysis of OS, baseline AFP ≥ 400 ng/ml and initial tumor shape irregularity were found to be significant independent prognostic factors for OS after adjusting for potential confounders (AFP ≥ 400 ng/ml, HR 1.73, 95% CI 1.01–2.96, *P* = 0.046; Initial tumor shape irregularity, HR 1.94, 95% CI 1.03–3.65, *P* = 0.041; Table 2). The irregular shape of the tumor is a significant predictor of progression-free survival based on the multivariate analysis (HR 2.15, 95% CI 1.40–3.29, *P* < 0.001).

**Table 2:** Results of univariate and multivariate Cox regression analysis of OS and PFS in the training cohort

Variables	Univariable		Multivariable	
	HR (95% CI)	*P* value	HR (95% CI)	*P* value
*Univariate and multivariate Cox regression analysis of OS*				
Age, years	0.26 (0.02–4.16)	0.338		
SEX, male vs. female	1.75 (0.96–3.20)	0.067	1.81 (0.86–3.79)	0.117
Hepatitis B virus, absent vs. present	0.84 (0.42–1.69)	0.629		
AST, IU/l	1.01 (1.01–1.01)	0.009	1.00 (1.00–1.00)	0.965
ALT, IU/l	1.00 (1.00–1.00)	0.157		
PLT, 10^9^/l	1.00 (1.00–1.00)	0.433		
WBC, 10^9^/l	1.00 (0.99–1.02)	0.766		
ECOG PS, 0 vs. ≥ 1	1.57 (0.97–2.55)	0.067	1.14 (0.65–2.00)	0.641
Prior TACE, absent vs. present	1.04 (0.66–1.63)	0.879		
Immunotherapy line, 1st- vs. 2nd-/later line	0.76 (0.47–1.23)	0.258		
Combined targeted therapy, absent vs. present	0.96 (0.61–1.51)	0.861		
Child–Pugh stage, A vs. B/C	1.23 (0.75–2.05)	0.413		
Tumor number, single vs. multiple	1.33 (0.70–2.53)	0.381		
Tumor size, < 5 vs. ≥ 5 cm	2.56 (1.49–4.42)	< 0.001	1.82 (0.94–3.52)	0.076
Lymph node metastasis, absent vs. present	1.59 (1.02–2.50)	0.043	1.25 (0.68–2.29)	0.467
Macrovascular invasion, absent vs. present	2.88 (1.80–4.60)	< 0.001	1.71 (0.88–3.33)	0.115
Extrahepatic spread, absent vs. present	1.06 (0.67–1.67)	0.799		
BCLC stage, 0/A/B vs. C	2.52 (1.29–4.91)	0.007	2.41 (0.83–6.96)	0.104
Cirrhosis, absent vs. present	0.65 (0.40–1.07)	0.090	0.96 (0.49–1.88)	0.912
Initial tumor shape, Regularity vs. Irregularity	1.89 (1.17–3.04)	0.009	1.94 (1.03–3.65)	0.041
Alpha-fetoprotein, < 400 vs. ≥ 400 ng/ml	1.88 (1.20–2.95)	0.006	1.73 (1.01–2.96)	0.046
*Univariate and multivariate Cox regression analysis of PFS*				
Age, years	0.99 (0.97–1.00)	0.169		
SEX, male vs. female	0.76 (0.40–1.47)	0.420		
Hepatitis B virus, absent vs. present	1.16 (0.59–2.30)	0.667		
AST, IU/l	1.00 (1.00–1.00)	0.293		
ALT, IU/l	1.00 (1.00–1.00)	0.737		
PLT, 10^9^/l	1.00 (1.00–1.00)	0.816		
WBC, 10^9^/l	1.01 (0.99–1.02)	0.522		
ECOG PS, 0 vs. ≥ 1	1.49 (0.99–2.23)	0.055	1.57 (1.04–2.37)	0.031
Prior TACE, absent vs. present	1.35 (0.93–1.98)	0.117		
Immunotherapy line, 1st- vs 2nd-/later line	0.81 (0.55–1.20)	0.299		
Combined targeted therapy, absent vs. present	1.04 (0.71–1.52)	0.852		
Child–Pugh stage, A vs. B/C	1.02 (0.66–1.57)	0.942		
Tumor number, single vs. multiple	1.21 (0.75–1.96)	0.428		
Tumor size, < 5 vs. ≥ 5 cm	0.96 (0.60–1.55)	0.875		
Lymph node metastasis, absent vs. present	1.39 (0.95–2.02)	0.090	1.26 (0.86–1.86)	0.240
Macrovascular invasion, absent vs. present	1.45 (0.98–2.15)	0.065	0.99 (0.65–1.51)	0.950
Extrahepatic spread, absent vs. present	0.91 (0.62–1.33)	0.625		
BCLC stage, 0/A/B vs. C	1.19 (0.77–1.85)	0.437		
Cirrhosis, absent vs. present	0.81 (0.51–1.28)	0.364		
Initial tumor shape, Regularity vs. Irregularity	2.19 (1.47–3.25)	< 0.001	2.15 (1.40–3.29)	< 0.001
Alpha-fetoprotein, < 400 vs. ≥ 400 ng/ml	1.49 (1.01–2.18)	0.043	1.25 (0.84–1.87)	0.269

*ECOG PS* Eastern Cooperative Oncology Group Performance Status, *BCLC* Barcelona-Clinic Liver Cancer, *TACE* transcatheter arterial chemoembolization, *ALT* alanine aminotransferase, *AST* aspartate aminotransferase, *PLT* platelet count, *WBC* white blood cell count, *NLR* neutrophil–lymphocyte ratio, *HR* hazard ratio

**Fig. 2:**
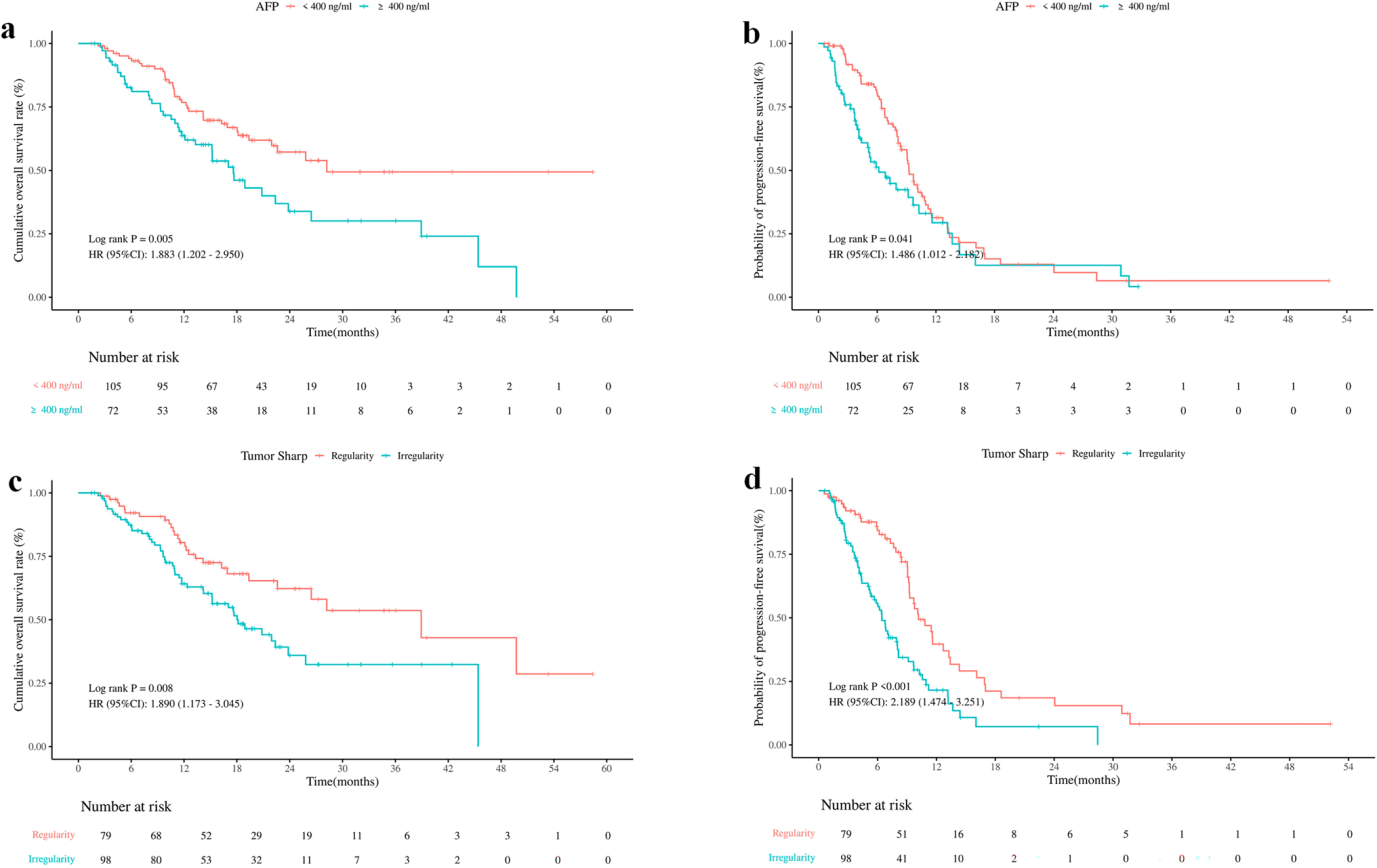
OS and PFS survival curves for AFP and irregular tumor shape. **a** Overall survival and **b** progression-free survival for baseline AFP level. **c** Overall survival and **d** progression-free survival for the irregular tumor shape

AFP ≥ 400 ng/ml and the initial tumor shape irregularity were both identified as significant prognostic factors for OS. In addition, the hazard ratios (HRs) for both factors were similar in multivariate analyses. Therefore, we devised a simple score for forecasting prognosis based on these two variables, named ATSI (AFP and Tumor Shape Irregularity). One point was assigned to each AFP ≥ 400 ng/ml or irregular initial tumor shape, thus dividing patients into three groups: ATSI score 0 points (AFP < 400 ng/ml and initial tumor shape regularity); ATSI score 1 point (AFP ≥ 400 ng/ml or initial tumor shape irregularity); ATSI score 2 points (AFP ≥ 400 ng/ml and initial tumor shape irregularity). Furthermore, the patients with higher AFP levels exhibited notably inferior median OS and PFS outcomes compared to those with lower AFP levels [median OS, 17.60 (95% CI 13.30–26.43) vs. 28.20 (95% CI 21.93–NA) months, *P* = 0.005; median PFS, 6.17 (95% CI 5.03–11.60) vs. 9.23 (95% CI 8.37–10.93) months, *P* = 0.041]. The median OS and PFS of patients with regular tumor shape were 38.93 months (95% CI 26.43–NA) and 10.17 (95% CI 9.23–13.40) months, respectively, which were significantly superior to those with irregular shapes [median OS, 18.13 months (95% CI 15.17–25.80), *P* = 0.008; median PFS, 6.43 months (95% CI 5.33–8.13), *P* < 0.001]. OS and PFS survival curves for AFP and initial tumor shape irregularity are shown in Fig. 2a–d.

### Objective efficacy and survival analysis in the training set

The complete follow-up imaging was available for 166 (93.79%) patients in the training set. 77 (43.50%) patients reached the endpoint of death. 110 (62.15%) patients with confirmed progressive disease at final analysis. In the training set, the median OS was 22.6 months (95% CI 18.13–NA), while the median PFS was 9.07 months (95% CI 7.87–10.10, Table 3). At the time of analysis, the best radiological response was as follows: CR, *n* = 13 (7.83%); PR, *n* = 21 (12.65%); SD, *n* = 91 (54.82%); and PD, *n* = 41 (24.70%) (Table 3). As a result, 125 patients achieved disease control with the disease control rate (DCR) of 75.30% and 41 patients achieved objective remission with the objective response rate (ORR) of 24.70%.

**Table 3:** Objective efficacy and survival analysis according to ATSI score in the training set

	Total (*n* = 177)	ATSI score 0 points *N* = 53	ATSI score 1 point *N* = 77	ATSI score 2 points *N* = 47	*P* value
Overall survival					0.001
Median (95% CI), months	22.60 (18.13–NA)	NA (28.20–NA)	25.80 (14.17–NA)	17.03 (11.73–23.83)	
Progression-free survival					< 0.001
Median (95% CI), months	9.07 (7.87–10.10)	10.83 (9.27–14.33)	8.03 (6.77–10.57)	5.03 (3.83–9.67)	
Best radiological response					< 0.001
Complete/partial response	34 (20.48)	8 (16.33)	20 (27.40)	6 (13.64)	
Stable disease	91 (54.82)	35 (71.43)	39 (53.42)	17 (38.64)	
Progressive disease	41 (24.70)	6 (12.24)	14 (19.18)	21 (47.73)	
Disease control ratio					< 0.001
Yes (CR/PR/SD)	125 (75.30)	43 (87.76)	59 (80.82)	23 (52.27)	
No (PD)	41 (24.70)	6 (12.24)	14 (19.18)	21 (47.73)	

*CR* complete response, *PD* progressive disease, *PR* partial response, *SD* stable disease

### Efficacy and survival prediction according to ATSI score in the training set

**Fig. 3:**
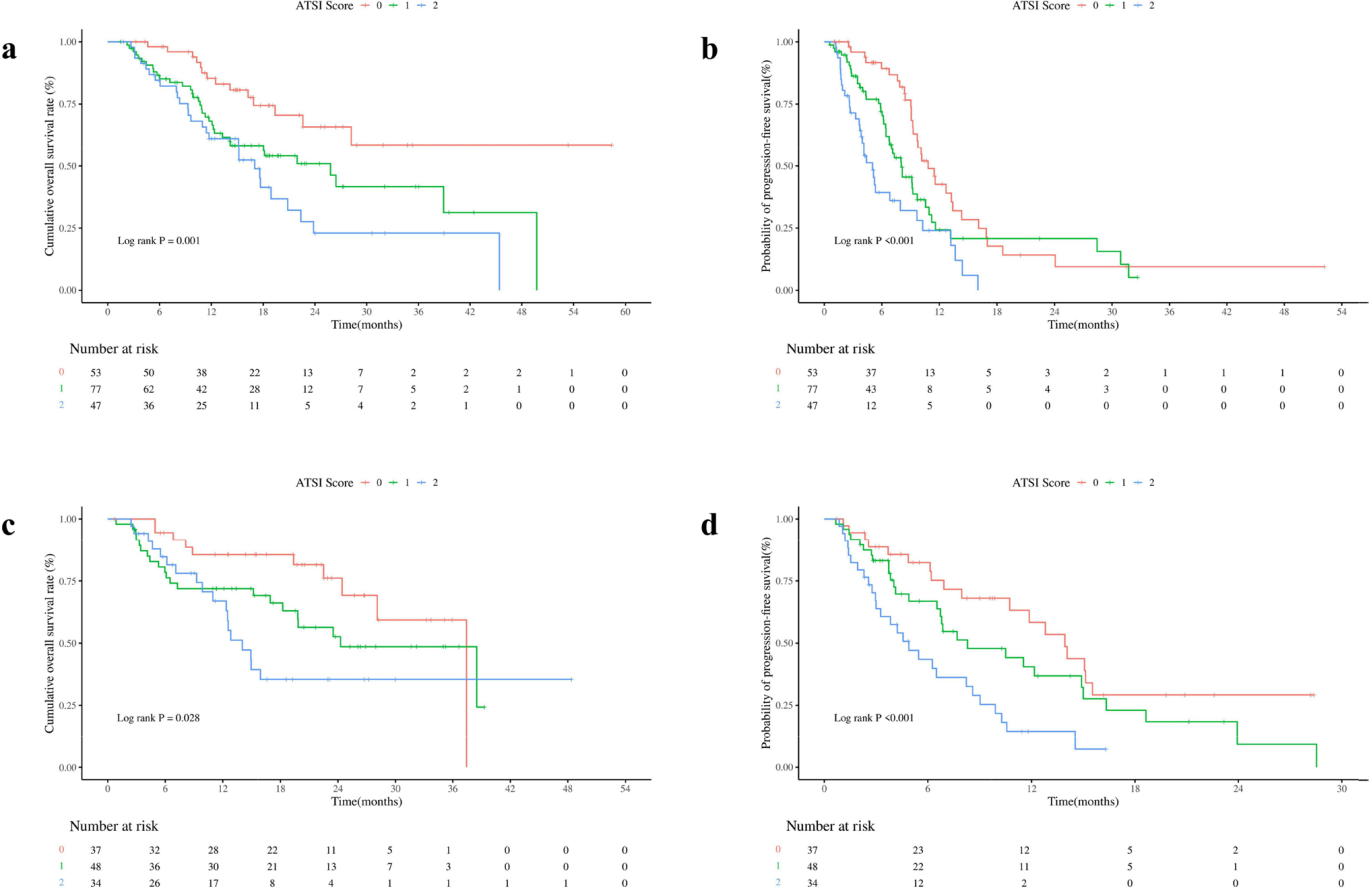
Kaplan–Meier curves for the training and validation sets according to ATSI score. **a** Overall survival and **b** progression-free survival for the training set according to ATSI score. **c** Overall survival and **d** progression-free survival for the validation set according to ATSI score

The training set cohort was stratified according to ATSI scores, resulting in 53 (29.94%) patients with 0 points, 77 (43.50%) patients with 1 point, and 47 (26.56%) patients with 2 points. The 1-year OS rate was 71.70%, 54.55%, and 53.19% in 0 points, 1 point, and 2 points (*P* = 0.090, Fig. 3a), respectively. The median OS was not reached in patients with 0 points (95% CI 28.20–NA), and the median OS was 25.80 (95% CI 14.17–NA) months in patients with 1 point, 17.03 (95% CI 11.73–23.83) months in patients with 2 points (*P* = 0.001, Table 3). In the training set, the median PFS was 10.83 (95% CI 9.27–14.33) months for ATSI score 0 points, 8.03 (95% CI 6.77–10.57) months for ATSI score 1 point, and 5.03 (95% CI 3.83–9.67) months for ATSI score 2 points (*P* < 0.001, Fig. 3b).

The best radiological response for each group after stratification according to ATSI score was as follows: CR was *n* = 3/49 (6.12%) vs. *n* = 8/73 (10.96%) vs. *n* = 2/44 (4.55%), and PR was *n* = 5/49 (10.20%) vs. *n* = 12/73 (16.44%) vs. *n* = 4/44 (9.09%) for ATSI-0 points vs. ATSI-1 point vs. ATSI-2 points, respectively; SD was *n* = 35/49 (71.43%) vs. *n* = 39/73 (53.42%) vs. *n* = 17/44 (38.64%), and PD was *n* = 6/49 (12.24%) vs. *n* = 14/73 (19.18%) vs. *n* = 21/44 (47.73%) for ATSI-0 points vs. ATSI-1 point vs. ATSI-2 points, respectively. DCR was 87.76%, 80.82%, and 52.27% in the 0 points, 1 point, and 2 points groups, respectively (*P* < 0.001, Table 3).

### Efficacy and survival prediction according to ATSI score in the validation set

The validation set included 119 HCC patients who received immunotherapy, of which 116 (97.48%) patients had complete imaging data. The best radiological response results in the validation cohort at the final analysis showed 9 (7.76%) patients in CR, 18 (15.52%) patients in PR, 59 (50.86%) patients in SD and 30 (25.86%) patients in PD (Table 4). ORR was 23.28% in the validation set, while DCR was 74.14%. The progressive disease was confirmed in 74 (62.18%) patients, with the median PFS of 8.30 months (95% CI 6.73–11.87, Table 4). 49 (41.18%) patients died at the final analysis, with the median OS of 28.10 months (95% CI 19.83–NA, Table 4).

**Table 4:** Objective efficacy and survival analysis according to ATSI score in the validation set

	Total (*n* = 119)	ATSI score 0 points *N* = 37	ATSI score 1 point *N* = 48	ATSI score 2 points *N* = 34	*P* value
Overall survival					0.028
Median (95% CI), months	28.10 (19.83–NA)	37.43 (28.10–NA)	24.27 (18.23–NA)	14.03 (12.37–NA)	
Progression-free survival					< 0.001
Median (95% CI), months	8.30 (6.73–11.87)	13.93 (10.73–NA)	8.30 (6.73–16.33)	4.90 (3.00–9.03)	
Best radiological response					0.060
CR/PR	27 (23.28)	10 (27.78)	11 (23.91)	6 (17.65)	
SD	59 (50.86)	21 (58.33)	25 (54.35)	13 (38.24)	
PD	30 (25.86)	5 (13.89)	10 (21.74)	15 (44.12)	
Disease control ratio					0.011
Yes (CR/PR/SD)	86 (74.14)	31 (86.11)	36 (78.26)	19 (55.88)	
No (PD)	30 (25.86)	5 (13.89)	10 (21.74)	15 (44.12)	

*CR* complete response, *PD* progressive disease, *PR* partial response, *SD* stable disease

The results of the ATSI score were as follows: ATSI-0 points, *n* = 37 (31.09%); ATSI-1 point, *n* = 48 (40.34%); and ATSI-2 points, *n* = 34 (28.57%). Kaplan–Meier survival curves according to ATSI score showed significant differences in PFS and OS among the three groups of patients (Fig. 3c, d). The median OS for patients with 0 points was 37.43 (95% CI 28.10–NA) months, which was significantly better than patients with 1 point at 24.27 (95% CI 18.23–NA) months and patients with 2 points at 14.03 (95% CI 12.37–NA) months (*P* = 0.028). The median PFS was 13.93 (95% CI 10.73–NA) months for 0 points, 8.30 (95% CI 6.73–16.33) months for 1 point and 4.90 (95% CI 3.00–9.03) months for 2 points (Table 4).

In the validation set, CR or PR was achieved in 10 (27.78%) patients with ATSI-0 points, in 11 (23.91%) patients with ATSI-1 point, and 6 (17.65%) patients with ATSI-2 points. Among the patients with stable diseases, there were 21 (58.33%) with 0 points, 25 (54.35%) with 1 point, and 13 (38.24%) with 2 points. 5 (13.89%) patients with 0 points, 10 (21.74%) with 1 point, and 15 (44.12%) with 2 points achieved progressive disease. The DCR was 86.11% for 0 points, 78.26% for 1 point, and 55.88% for 2 points, respectively (*P* = 0.011, Table 4).

### Objective response analysis after adjustment in the training and validation sets

Considering that some patients had incomplete follow-up imaging (6.21% in the training set; 2.52% in the validation set), we analyzed them according to the worst-case scenario and assigned all patients with missing follow-up imaging data to the PD group and the differences in DCR between the groups stratified by the ATSI score were still statistically significant (Table S1). Based on this, the ATSI score was effective in predicting tumor response in HCC patients receiving immunotherapy.

In the multiple comparisons between each group, we found that the differences in best radiological response and DCR were not statistically significant in those ATSI-0 points versus ATSI-1 point in both the training and validation sets (*P* > 0.05). However, the radiological response and DCR were significantly superior in both patients with ATSI-0 points and ATSI-1 point than ATSI-2 points in both the training and validation sets (*P* < 0.05).

### Efficacy and survival prediction according to ATSI score in the overall population

Additionally, we have tested the effects of AFP and tumor shape irregularity in the overall population. The results showed that patients with AFP ≥ 400 ng/ml had inferior OS and PFS than those with AFP < 400 ng/ml (*P* < 0.05, Fig. S3ab) and patients with irregularly shaped tumors also had significantly worse OS and PFS than those with regular shapes (*P* < 0.001, Fig. S3cd). Subsequently, we tested the efficacy of the ATSI score in the overall population, both the OS and PFS in patients with 0 points were the longest, followed by patients with 1 point, and patients with 2 points had the worst OS and PFS (*P* < 0.001, Fig. S4). In addition, we analyzed the impact of prior TACE therapy and first-line or second/later-line immunotherapy on patients’ survival. The results showed that the predictive performance of the ATSI score is well represented in both first-line and second/later-line immunotherapy populations (Fig. S5). Similarly, the ATSI score showed a good predictive performance in the population with or without prior TACE treatment (Fig. S6). Furthermore, we found that the ATSI score also showed a favorable predictive effect in both early/intermediate-stage and advanced-stage HCC patients (*P* < 0.05, Fig. S7).

Of the 184 patients with tumor progression, 111 patients were treated with subsequent immunotherapy after tumor progression, whereas targeted therapy was applied to 65 patients, and 56 patients were treated with targeted therapy in combination with immunotherapy. The survival analysis shows that patients who received either immunotherapy alone or targeted therapy combined with immunotherapy had better OS than those who did not receive subsequent therapy (median OS, 37.43 (95% CI 18.13–NA) vs. 28.10 (95% CI 19.80–NA) vs. 14.97 (95% CI 10.87–26.43) months, *P* = 0.002, Fig. S8). However, there was no statistically significant difference in the prognosis between patients who received immunotherapy alone and the patients who received targeted therapy combined with immunotherapy after progression (*P* > 0.05, Fig. S8).

Albumin-bilirubin (ALBI) grade has been proposed and validated to predict survival benefits for HCC patients receiving immunotherapy, we compared the area under the curve (AUC) and net reclassification improvements (NRI) in predictive performance between ATSI score and ALBI grade (Table S2). The results showed that the ATSI score had higher AUC values compared to the ALBI grade in terms of discriminating survival outcomes, suggesting that the ATSI score has a better discriminatory ability in predicting OS of HCC patients receiving immunotherapy (AUC: 0.627 vs. 0.546, *P* = 0.039, Fig. S9). In addition, although not statistically significant, continuous NRI analysis showed improved performance of ATSI score compared to ALBI grade, with a 19.8% increase in the percentage of correct predictions [continuous NRI (95% CI): 0.198 (− 0.031 to 0.294), *P* = 0.096]. We believe that this may be due to the relatively small sample size of our study and the specific characteristics of the patient population.

## Discussion

In the multivariate analysis, we found that the baseline AFP ≥ 400 ng/ml and initial tumor shape irregularity were both significant independent factors predicting the prognosis of patients with HCC undergoing immunotherapy. Furthermore, we constructed a simple and reliable scoring system named ATSI score to forecast the efficacy and prognosis of HCC patients undergoing immunotherapy. The stratification of the ATSI score showed significant differences in OS, PFS and DCR in the training set, which further verified its utility in the validation set.

Multiple research studies have indicated that the current biomarkers for predicting the efficacy of ICIs in HCC include PD-L1 expression level, TMB, circulating tumor DNA, dMMR and MSI [10, 25]. However, PD-L1 expression did not significantly relate to the anti-tumor efficacy in HCC patients treated with nivolumab [13]. Furthermore, previous studies have demonstrated that TMB could predict tumor response in patients treated with pembrolizumab [26], but fewer HCC patients with high TMB [26, 27]. Most studies of circulating tumor DNA or circulating tumor cells are small-sample, retrospective analyses with insufficient strength of evidence to assess clinical translational value [28, 29]. Besides, the drawbacks such as cumbersome sampling and assay methods, high cost, and poor timeliness make it difficult to test these biomarkers on a large scale in a clinical setting [13–15]. Therefore, the utility of these markers in real clinical work is low.

In addition to being a diagnostic biomarker for HCC, AFP is highly correlated with the malignant outcomes of HCC patients and the malignant biological behavior of HCC cells. Prior research has shown that high AFP levels may indicate tumor progression. The activation of growth signaling and the inhibition of apoptotic signaling by AFP promote cancer cell proliferation and metastasis. Additionally, AFP assists tumors in escaping immune attack and promotes HCC cell growth [30]. The high levels of AFP expression can cause dendritic cell (DC) dysfunction or even apoptosis, which affects T lymphocyte differentiation and alters the CD4 + /CD8 + T cell ratio, subsequently impacting the level of T cell immune response [31, 32]. AFP correlates with several tumor target proteins for immunotherapy, including VEGFR and epithelial cell adhesion molecule (EpCAM) [33]. Based on this, HCC patients with elevated AFP may have a poorer response to immunotherapy. The results from several studies suggest that higher AFP levels are associated with a favorable predictive value for the prognosis of immunotherapy in patients with HCC [16, 17]. Therefore, AFP levels are considered a clinically sensitive indicator of treatment efficacy [34] and AFP ≥ 400 ng/ml indicate unfavorable prognosis in various clinical situations [35–37], which is consistent with our results.

Based on this, the combination of AFP with peripheral blood biomarkers has been explored in many studies to provide a more accurate scoring system. Scheiner B et al. developed the CLAFITY score, which combines AFP and C-reactive protein (CRP), and retrospective analysis showed that this score was effective in predicting efficacy and prognosis in patients undergoing PD-(L)1-targeted immunotherapy [16]. Furthermore, Hatanaka T et al. combined AFP with modified ALBI grade to forecast the prognosis of individuals with HCC undergoing treatment with atezolizumab and bevacizumab [17]. Another study shows that the combination of AFP and neutrophil-to-lymphocyte ratio (NLR) can be utilized to forecast survival results and effectiveness in individuals undergoing ICIs [38]. However, due to inflammation and infection, peripheral blood biomarkers are often unstable. Therefore, more clinically useful and stable prognostic factors for immunotherapy are needed, especially imaging of tumors.

With the development of radiology, tumor imaging features have become reliable imaging biomarkers for forecasting the effectiveness and prognosis of immunotherapy in HCC patients. Yuan et al. developed and validated a radiomics nomogram using contrast-enhanced CT scans with AUC of 0.883 in the validation group, which showed that a blend of HCC imaging histologic characteristics and clinical factors could forecast the outcomes of immunotherapy in Chinese HCC patients [39]. Furthermore, Xu et al. established an MRI-based nomogram showing that MRI features of patients with unresectable HCC on treatment with combined lenvatinib and anti-PD-1 antibody therapy were effective in predicting disease progression and prognosis [40]. In addition, Sun et al. found that among patients with advanced HCC who received sintilimab in combination with IBI305, the contrast-enhanced ultrasound-based nomogram was effective in predicting disease progression [41]. Radiology has great potential for predicting prognosis and assessing efficacy of HCC immunotherapy, and imaging scoring models that incorporate clinical risk factors have superior predictive performance [39–41]. But the reproducibility of HCC tumor imaging characteristics is reduced when studies are performed on different CT or MRI platforms, and imaging sequences lack standardization and consistency [42]. The imaging histology process is complex, time-consuming, and requires specialized software, making clinical application difficult and underutilized. As a result, there is a lack of simple, clinical judgements of generalizability of imaging indices.

As we know, depending on the CT and MRI manifestations of HCC, the tumor shape may appear regular or irregular. Previous studies have found that the high correlation between irregular tumor shape and the preoperative diagnosis and location of microvascular invasion (MVI) [43, 44]. In addition, previous studies have significantly associated irregular tumor shape with severe MVI, and the 5-year OS and recurrence free survival (RFS) rates were inferior in HCC patients with irregular tumor shape than in HCC patients with regular tumor shape [18]. Consistent with our findings, irregular tumor shape was closely related to poorer PFS and OS in HCC patients undergoing immunotherapy. But it is important to know how to define whether a tumor is regular or irregular and what indicators need to be considered. First, tumor margins are the regions where tumor cells invade surrounding tissues and directly interact with other cells and where tumor cell infiltration and invasion are most pronounced [21], leading to significant immunosuppressive effects [22]. Furthermore, the irregular shape of the tumor can be taken as an indication of the aggressive biological tendency to infiltrate the tumor boundary and extend into the surrounding non-tumor tissue [23]. However, limited research has examined the impact of irregular tumor shape on the efficacy and prognosis of immunotherapy in HCC patients. During this study across multiple centers, we discovered that irregular tumor shape was highly related to inferior OS, PFS and DCR in HCC patients undergoing ICIs. Based on this, we combined AFP with tumor shape irregularity to develop and validate the ATSI score for predicting survival and tumor response to immunotherapy. The ATSI score is stable, simple, and has high clinical utility and significant predictive performance.

About 11% of patients with HCC of BCLC Stage 0 or A were enrolled in this study. According to the guidelines, curative resection, including surgery and ablation, is recommended for such patients. The main reasons why they receive immunotherapy rather than radical treatment are that this group of patients is unable to undergo surgery after tumor recurrence due to insufficient residual liver volume, and the tumors were adjacent to major vessels and other organs that cannot perform ablation, or the patients themselves refuse to undergo curative treatment again.

Nevertheless, there are some limitations of this study. First, this is a retrospective multi-center study and the relatively small sample size of this study makes statistical inferences of limited validity, which may bias the results obtained. Although the level of evidence is not as high as prospective external validation, but it is an internal randomized validation, replication of results and the detection of performance for this score are still reliable and meaningful. Second, the patients receiving immunotherapy included in this study were all in China, and the vast majority of them were infected with hepatitis B, which may differ from the baseline characteristics of the western populations. Based on the above, further prospective and larger sample cohorts are necessary to validate the predictive effect of the ATSI score on immunotherapy in HCC patients.

In conclusion, we developed a simple and reliable scoring system to predict the efficacy and prognosis of immunotherapy in HCC patients based on two easily accessible clinic metrics: baseline AFP and initial tumor shape. The ATSI score can effectively screen the population for immunotherapy benefits. The effect needs to be subsequently confirmed in large prospective clinical studies.

## Conclusion

The easily applicable ATSI score based on the baseline AFP level and initial tumor shape can effectively predict efficacy and survival in HCC patients with ICIs. 

## Supplementary Information

Below is the link to the electronic supplementary material.

Supplementary file1 (DOCX 2549 kb)

Supplementary file2 (DOCX 19 kb)

## Abbreviations

ICIs: Immune checkpoint inhibitors
HCC: Hepatocellular carcinoma
AFP: Alpha-fetoprotein
OS: Overall survival
PFS: Progression-free survival
DCR: Disease control rate
BMI: Body mass index
ECOG-PS: Eastern Cooperative Oncology Group Performance Status
ROC: Receiver operating characteristic
TACE: Transcatheter arterial chemoembolization
BCLC: Barcelona Clinic Liver Cancer
PD-L1: Programmed death ligand 1
dMMR: Deficient mismatch repair
TMB: Tumor mutational burden
CT: Computed tomography
MRI: Magnetic resonance imaging
CR: Complete response
PR: Partial response
SD: Stable disease
PD: Progressive disease
HR: Hazard ratio
ORR: Objective response rate
DC: Dendritic cell
EpCAM: Epithelial cell adhesion molecule
ALBI: Albumin-bilirubin
AUC: Area under the curve
CRP: C-reactive protein
MVI: Microvascular invasion
MSI: Microsatellite instability
RFS: Recurrence free survival
NLR: Neutrophil-to-lymphocyte ratio
