# Supplementary material for: Alpha-fetoprotein combined with initial tumor shape irregularity in predicting the survival of patients with advanced hepatocellular carcinoma treated with immune-checkpoint inhibitors: a retrospective multi-center cohort study
Source: J Gastroenterol. 2024 Dec 23;60(4):442–55. doi: 10.1007/s00535-024-02202-y (PMC11922967; doi:10.1007/s00535-024-02202-y)
Supplement: Supplementary file 1 — Supplementary file1 (DOCX 2549 kb) [file 535_2024_2202_MOESM1_ESM.docx]

***Alpha-fetoprotein combined with initial tumor shape irregularity in predicting the survival of patients with advanced hepatocellular carcinoma treated with immune-checkpoint inhibitors: a retrospective multi-center cohort study***

***Journal name: Journal of Gastroenterology***

Feng Zhang^123#^, Yong-Shuai Wang^123#^, Shao-Peng Li^4#^, Bin Zhao^123^, Nan Huang^123^, Rui-Peng Song^123^, Fan-Zheng Meng^123^, Zhi-Wen Feng^5^, Shen-Yu Zhang^123^, Hua-Chuan Song^123^, Xiao-Peng Chen^5*^, Lian-Xin Liu^123*^, Ji-Zhou Wang^123*^

**Corresponding Author:**

Ji-Zhou Wang

Department of Hepatobiliary Surgery, The First Affiliated Hospital of USTC, Division of Life Sciences and Medicine, University of Science and Technology of China, Hefei, Anhui, 230001, China.

Email: wangjoe@ustc.edu.cn

Tel: 86-13836135864

Orcid ID: 0000-0002-6934-072X





Fig. S1 Flow chart of the study

Note: HCC, hepatocellular carcinoma; ICIs, Immune checkpoint inhibitors.





Fig. S2 ROC curves for AFP based on OS

Note: ROC, Receiver operating characteristic curve; OS, Overall survival.


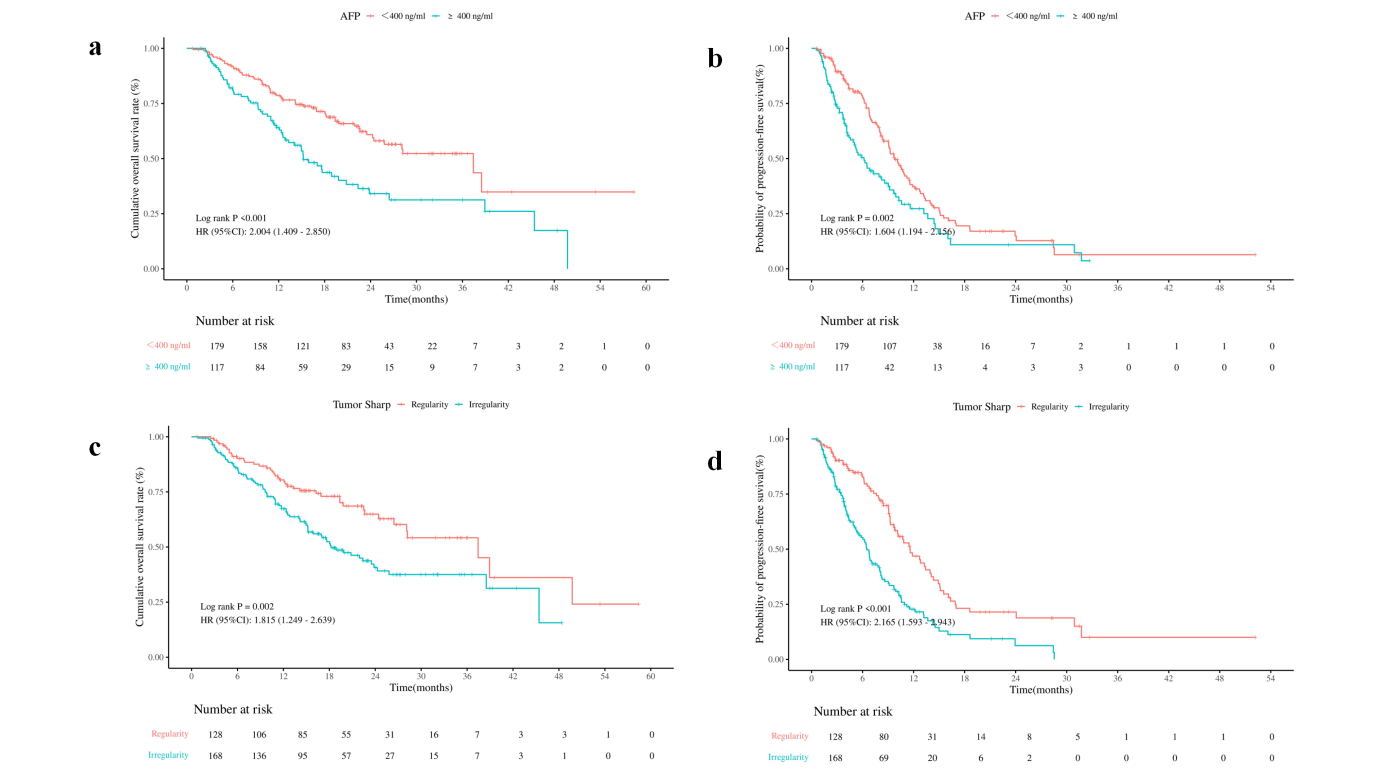


Fig. S3 OS and PFS survival curves for AFP and irregular tumor shape in the overall population

a. Overall survival and b. Progression-free survival for baseline AFP level. c. Overall survival and d. Progression-free survival for the irregular tumor shape.

Note: OS, Overall survival; PFS, Progression-free survival.


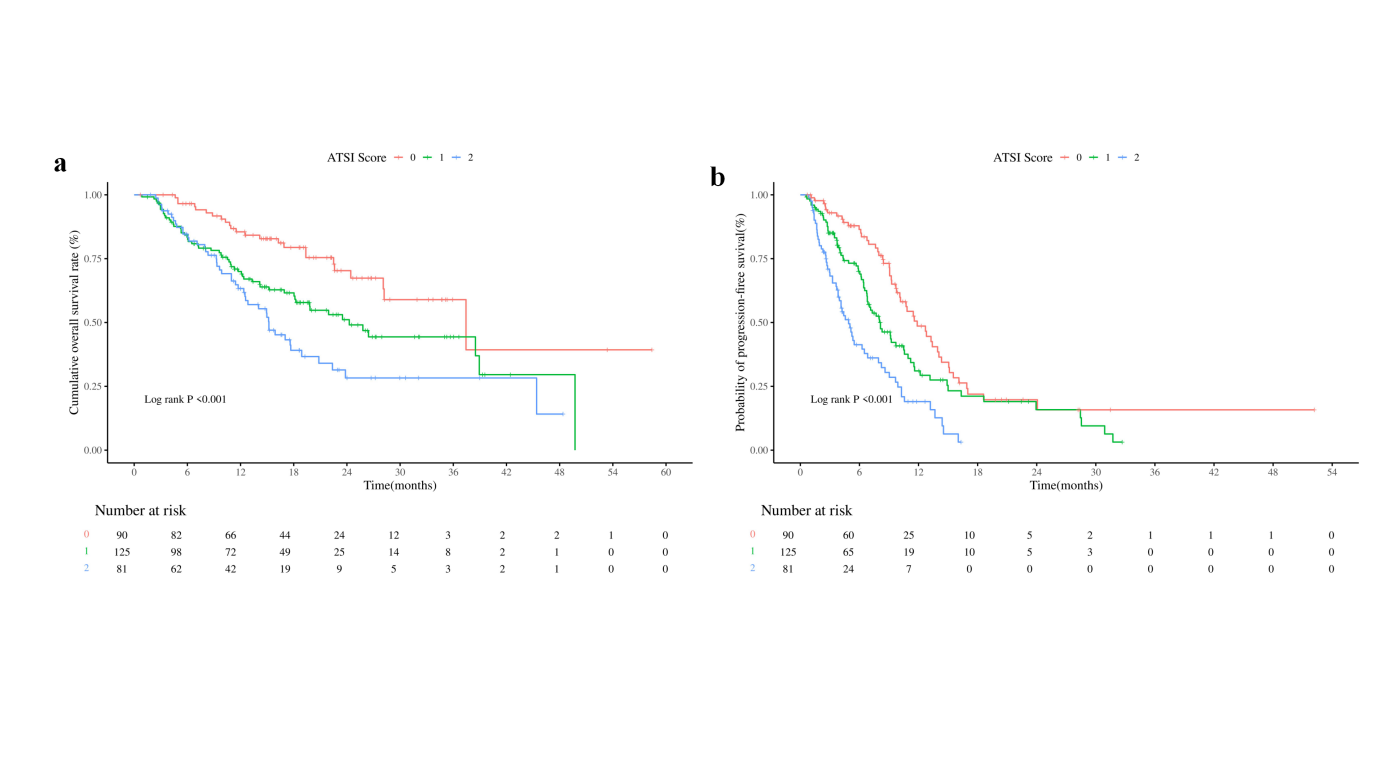


Fig. S4 Kaplan-Meier curves for the overall population according to ATSI score

a. Overall survival and b. Progression-free survival for the overall population according to ATSI score.

Fig. S5 Kaplan-Meier curves for the first-line and second/later-line immunotherapy populations according to ATSI score

a. Overall survival and b. Progression-free survival for the first-line immunotherapy population according to ATSI score. c. Overall survival and d. Progression-free survival for the second/later-line immunotherapy population according to ATSI score.

Fig. S6 Kaplan-Meier curves for the with or without prior TACE treatment populations according to ATSI score

a. Overall survival and b. Progression-free survival for the with prior TACE treatment population according to ATSI score. c. Overall survival and d. Progression-free survival for the without prior TACE treatment population according to ATSI score.

Note: TACE, transcatheter arterial chemoembolization.


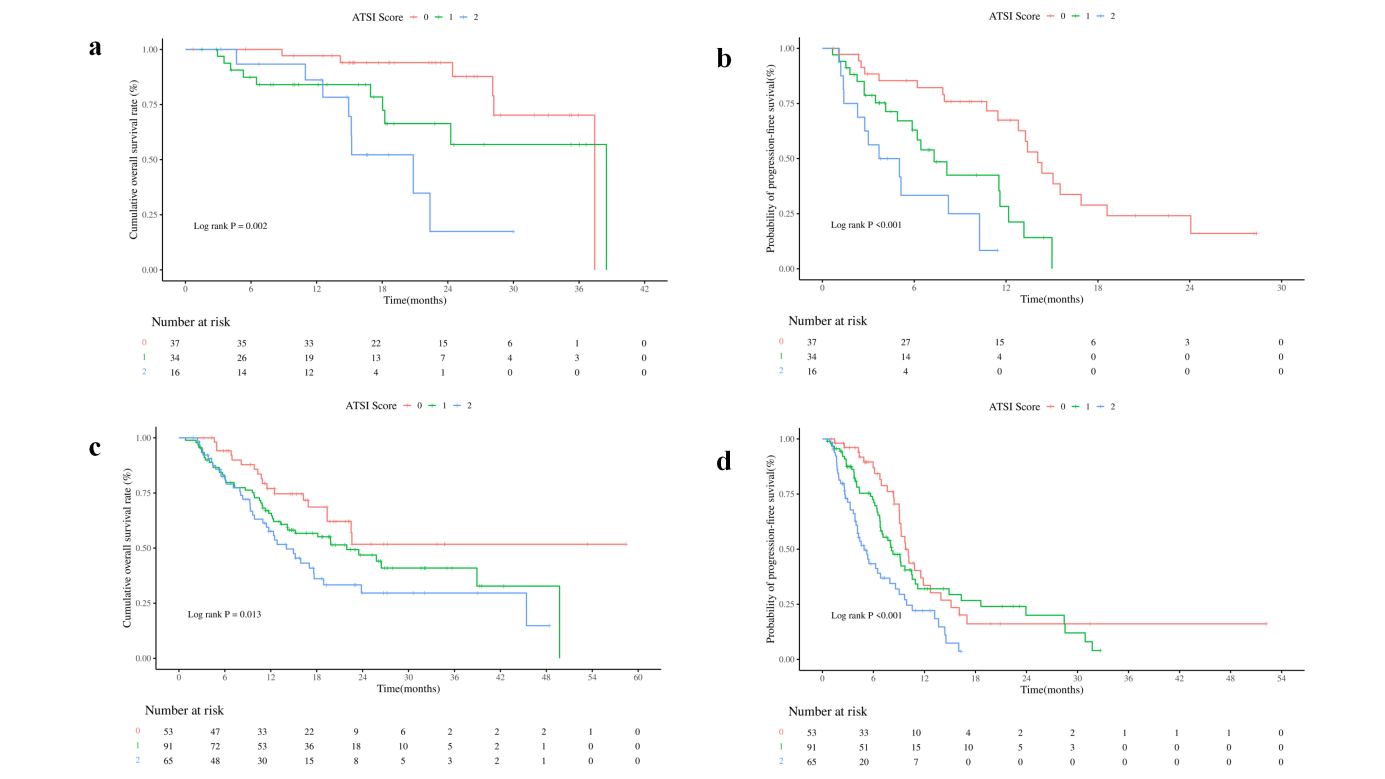


Fig. S7 Kaplan-Meier curves for the early/intermediate-stage and advanced-stage HCC populations according to ATSI score

a. Overall survival and b. Progression-free survival for the early/intermediate-stage HCC population according to ATSI score. c. Overall survival and d. Progression-free survival for the advanced-stage HCC population according to ATSI score.


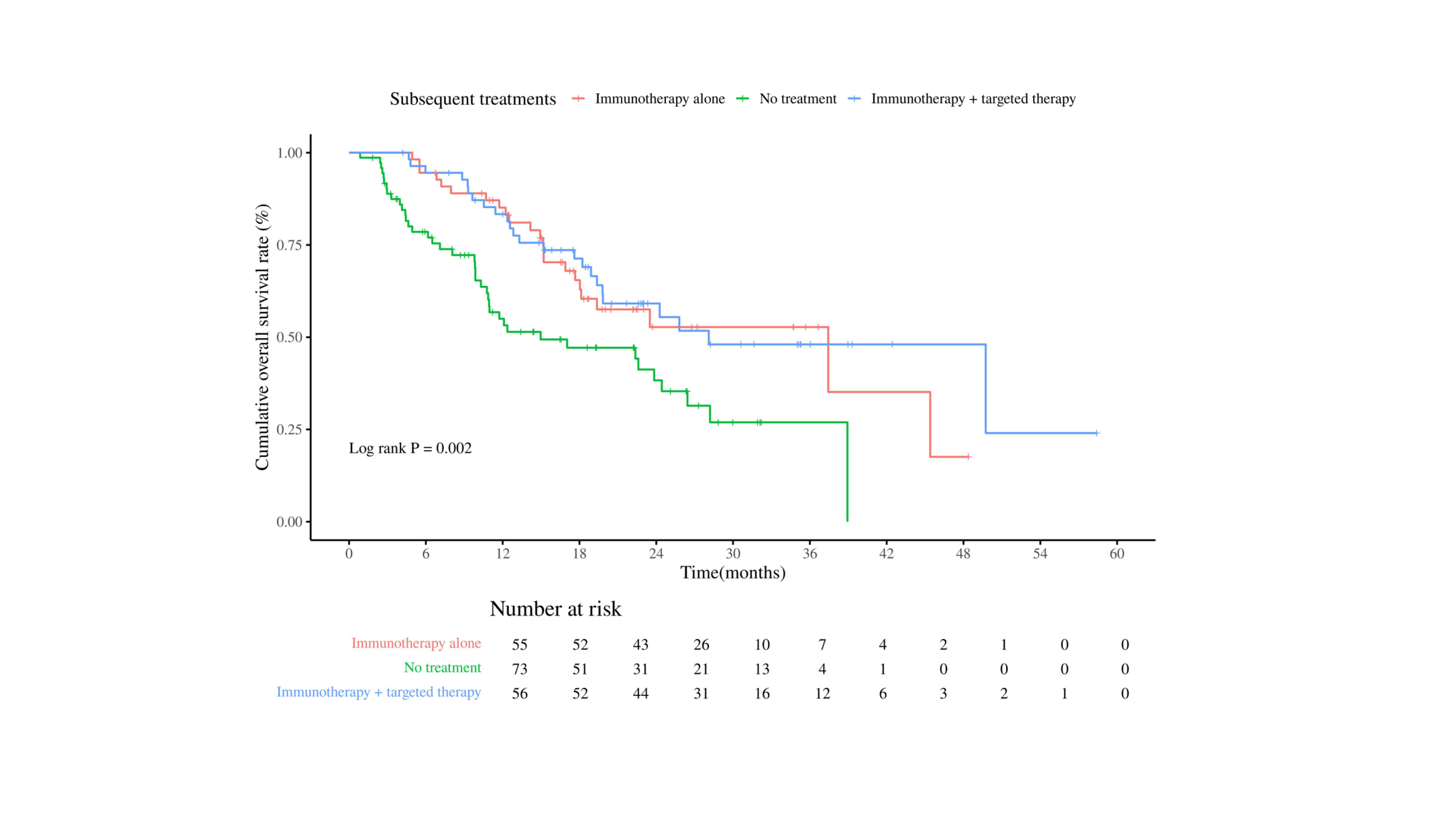


Fig. S8 Overall survival curves for subsequent treatments after tumor progression


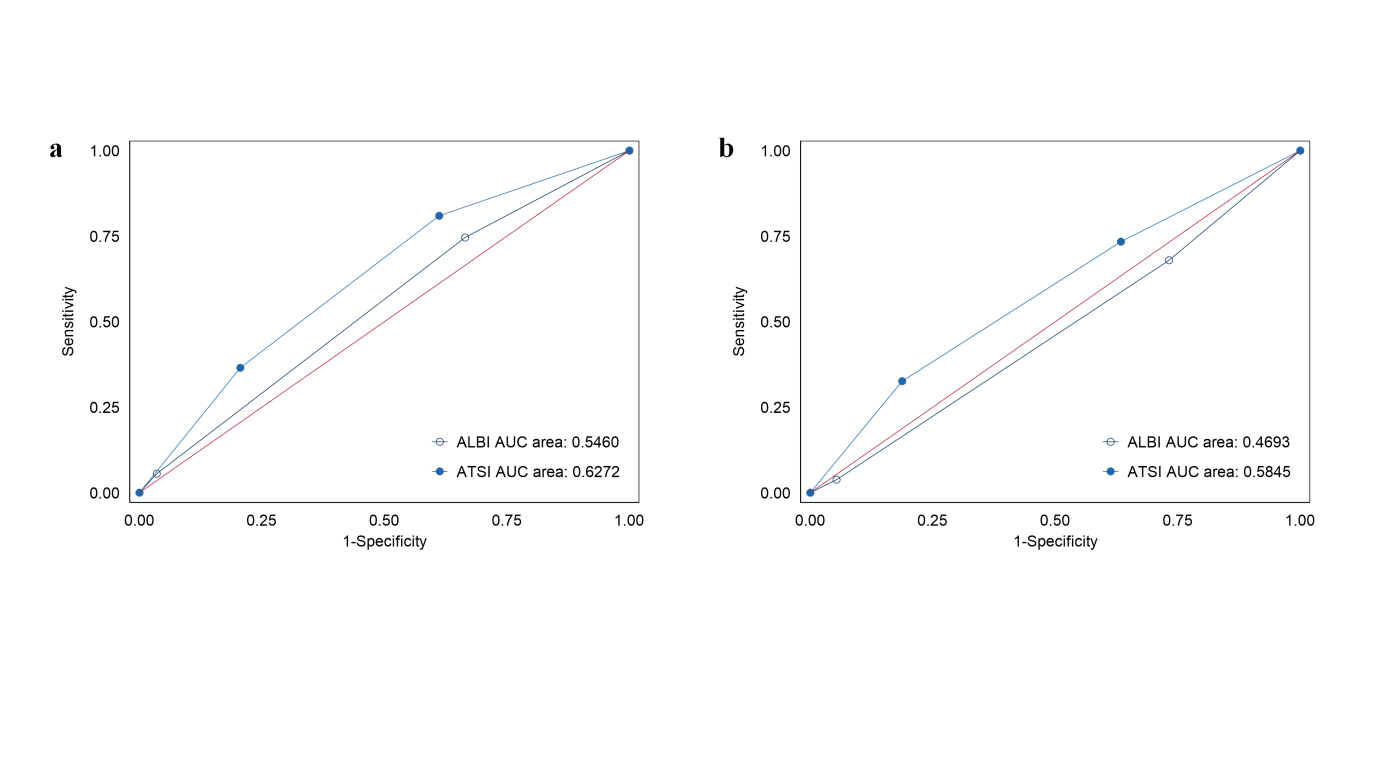


Fig. S9 ROC curves for ATSI score and ALBI grade based on OS and PFS

a. ROC curves for ATSI score and ALBI grade based on OS. b. ROC curves for ATSI score and ALBI grade based on PFS.

Note: ROC, Receiver operating characteristic curve; OS, Overall survival; PFS, Progression-free survival.
