# Supplementary material for: Alpha-fetoprotein combined with initial tumor shape irregularity in predicting the survival of patients with advanced hepatocellular carcinoma treated with immune-checkpoint inhibitors: a retrospective multi-center cohort study
Source: J Gastroenterol. 2024 Dec 23;60(4):442–55. doi: 10.1007/s00535-024-02202-y (PMC11922967; doi:10.1007/s00535-024-02202-y)
Supplement: Supplementary file 2 — Supplementary file2 (DOCX 19 kb) [file 535_2024_2202_MOESM2_ESM.docx]

***Alpha-fetoprotein combined with initial tumor shape irregularity in predicting the survival of patients with advanced hepatocellular carcinoma treated with immune-checkpoint inhibitors: a retrospective multi-center cohort study***

***Journal name: Journal of Gastroenterology***

Feng Zhang^123#^, Yong-Shuai Wang^123#^, Shao-Peng Li^4#^, Bin Zhao^123^, Nan Huang^123^, Rui-Peng Song^123^, Fan-Zheng Meng^123^, Zhi-Wen Feng^5^, Shen-Yu Zhang^123^, Hua-Chuan Song^123^, Xiao-Peng Chen^5*^, Lian-Xin Liu^123*^, Ji-Zhou Wang^123*^

**Corresponding Author:**

Ji-Zhou Wang

Department of Hepatobiliary Surgery, The First Affiliated Hospital of USTC, Division of Life Sciences and Medicine, University of Science and Technology of China, Hefei, Anhui, 230001, China.

Email: wangjoe@ustc.edu.cn

Tel: 86-13836135864

Orcid ID: 0000-0002-6934-072X

Table S1. Objective response analysis after adjustment in the training and validation sets

|  | ATSI score 0 points | ATSI score 1 point | ATSI score 2 points | *p* value |
| --- | --- | --- | --- | --- |
| Objective response analysis after adjustment in the training set | | | | |
| Best radiological response, n (%) |  |  |  | 0.006 |
| CR | 3 (5.66) | 8 (10.39) | 2 (4.26) |  |
| PR | 5 (9.43) | 12 (15.58) | 4 (8.51) |  |
| SD | 35 (66.04) | 39 (50.65) | 17 (36.17) |  |
| PD | 10 (18.87) | 18 (23.38) | 24 (51.06) |  |
| Disease control ratio, n (%) |  |  |  | <0.001 |
| Yes (CR/PR/SD) | 43 (81.13) | 59 (76.62) | 23 (48.94) |  |
| No (PD) | 10 (18.87) | 18 (23.38) | 24 (51.06) |  |
| Objective response analysis after adjustment in the validation set | | | | |
| Best radiological response, n (%) |  |  |  | 0.211 |
| CR | 3 (8.11) | 5 (10.42) | 1 (2.94) |  |
| PR | 7 (18.92) | 6 (12.50) | 5 (14.71) |  |
| SD | 21 (56.76) | 25 (52.08) | 13 (38.24) |  |
| PD | 6 (16.22) | 12 (25.00) | 15 (44.12) |  |
| Disease control ratio, n (%) |  |  |  | 0.028 |
| Yes (CR/PR/SD) | 31 (83.78) | 36 (75.00) | 19 (55.88) |  |
| No (PD) | 6 (16.22) | 12 (25.00) | 15 (44.12) |  |

Note: CR, complete response; PD, progressive disease; PR, partial response; SD, stable disease.

Table S2. Comparison of AUC and net reclassification improvement for predictive performance of ATSI score and ALBI grade

|  | AUC | *p* value | Continuous NRI (95%CI) | *p* value |
| --- | --- | --- | --- | --- |
| Predictive performance on OS | | | | |
| ALBI grade | 0.546 | Ref. |  | Ref. |
| ATSI score | 0.627 | 0.039 | 0.198 (-0.031 ~ 0.294) | 0.096 |
| Predictive performance on PFS | | | | |
| ALBI grade | 0.469 | Ref. |  | Ref. |
| ATSI score | 0.585 | 0.003 | 0.139 (-0.111 ~ 0.240) | 0.131 |

Note: AUC, area under the curve; NRI, net reclassification improvement.
